# Collagen adhesin protein and necrotic enteritis B-like toxin as biomarkers for early diagnosis of necrotic enteritis in commercial broiler chickens

**DOI:** 10.1016/j.psj.2023.102647

**Published:** 2023-03-14

**Authors:** D. Goo, I. Park, H. Nam, Y. Lee, J. Sawall, A.H. Smith, T.G. Rehberger, C. Li, H.S. Lillehoj

**Affiliations:** ⁎ Animal Bioscience and Biotechnology Laboratory, Beltsville Agricultural Research Center, Agricultural Research Service, USDA, Beltsville, MD, USA; † Department of Poultry Science, University of Georgia, Athens, GA, USA; ‡ Arm & Hammer Animal and Food Production, Waukesha, WI, USA

**Keywords:** broiler, collagen adhesin protein, necrotic enteritis, necrotic enteritis (NE) B-like toxin, sandwich ELISA

## Abstract

Mouse monoclonal antibodies (**mAbs**) reactive with *Clostridium perfringens* collagen adhesin protein (**CNA**) and necrotic enteritis B-like toxin (**NetB**) were developed. The best capture/detection mAb pairs for CNA and NetB were selected based on their affinity and specificity to develop sandwich enzyme-linked immunosorbent assays (**ELISAs**) to detect CNA and NetB proteins, respectively, in jejunal digesta samples from commercial broiler farms in the United States. Prior to the analysis of samples from commercial broiler flocks, the specificity and sensitivity of the CNA and NetB ELISAs were validated using sera, jejunal digesta, and fecal samples from chickens coinfected with *Eimeria maxima* and CNA^+^/NetB^+^*C. perfringens* in an animal model of necrotic enteritis (**NE**). Subsequently, a total of 251 field samples were collected from 74 commercial poultry farms. Among these, 18 samples were from 6 broiler farms that used certified organics (**CO**), and 155 samples were from 42 farms with nonantibiotics (**NA**). In jejunal digesta samples, CNA levels ranged from 0.02 to 0.59 ng/mL and NetB levels ranged from 0.09 to 1.91 ng/mL. CNA and NetB levels showed a positive correlation with each other (Pearson correlation coefficient *r* = 0.772, *P* < 0.001). CNA and NetB levels in jejunal digesta were significantly decreased in CO farms compared with those from NA farms (*P* < 0.001). In conclusion, these new *C. perfringens* antigen-specific sandwich ELISAs offer a sensitive and specific means to detect *C. perfringens* CNA and NetB proteins as biomarkers of early NE occurrence in field samples from commercial broiler chickens.

## INTRODUCTION

Necrotic enteritis (**NE**) is an enteric bacterial disease caused by anaerobic gram-positive toxicogenic *Clostridium perfringens*. Depending on their abilities to produce toxins, various strains (Type A–G) of *C. perfringens* have been designated based on virulence factors, which are implicated in several enterotoxic diseases in humans and animals (Rood et al., 2018). Among them, *C. perfringens* strain type G produces a variety of toxins including NE B-like toxin (**NetB**). Although other toxins (α-toxin, β-toxin, ε-toxin, ι-toxin, and enterotoxin) from *C. perfringens* are known to be involved in NE development, the major toxin implicated in the pathogenesis and development of NE in broilers is NetB produced by *C. perfringens* (Keyburn et al., 2010; Kiu and Hall, 2018; Lee and Lillehoj, 2021). These toxins have been associated with field NE disease outbreaks which globally cost economic losses of approximately $ 6 billion annually in the poultry industry (Wade and Keyburn, 2015). Nevertheless, there is limited information on the nature of *C. perfringens* involved in NE pathogenesis. A better understanding of pathogenic factors involved in host–pathogen interaction in NE will enable the development of effective strategies to prevent or mitigate NE.

In most bacterial diseases, the pathogenesis of infection begins in the initial stages when the invading bacteria adhere to host cell receptor (Reed and Williams Jr, 1978). Bacterial adhesins are specific structures that mediate attachment to host extracellular matrix (**ECM**) molecules, and dictate environmental tropism and successful colonization after attachment (Krogfelt, 1991; Klemm et al., 2007). Collagen, one of the essential components of ECM, can be a major attachment target of virulence factors produced by gram-positive pathogens (Martin and Smyth, 2010; Arora et al., 2021). If the intestinal epithelium of a chicken is damaged by various external factors such as coccidiosis, heat stress, litter conditions, or changed intestinal pH (Van Immerseel et al., 2004; Dahiya et al., 2006; Van Immerseel et al., 2009; Chen et al., 2019; Santos et al., 2019), ECM is likely to be exposed, raising the possibility that bacteria will easily attach to several collagen types (Lepp et al., 2021). Collagen adhesin protein (**CNA**) is the bacterial cell wall-anchored protein, and all CNA-like proteins are known to have properties that are directly attached to the host cell wall (Arora et al., 2021). Several gram-positive bacteria, such as *Staphylococcus aureus, Enterococcus faecalis*, and *Streptococcus mutans* also have binding capabilities in a similar manner to the role of CNA (Hubble et al., 2003; Sato et al., 2004; Xu et al., 2004). However, compared with extensive studies on CNA-like characteristics of these bacteria, literature on the role of CNA of *C. perfringens* in NE pathogenesis in chickens is still limited (Arora et al., 2021; Lepp et al., 2021). The adhesion ability of *C. perfringens* is known to be a key factor in NE pathogenesis. It is known that the pili of *C. perfringens* contains collagen-binding domains (Lepp et al., 2021). Several studies have shown that bacterial colonization is facilitated by the presence of the CNA of *C. perfringens* in the intestine of chickens (Wade et al., 2015; Ronco et al., 2017; Lacey et al., 2018). Furthermore, NE-causing isolates of *C. perfringens* are generally enriched in CNA-related genes compared to their healthy counterpart isolates, which supports that CNA is an important factor in NE pathogenesis (Kiu et al., 2019).

Despite the significant potential as a virulence factor, *C. perfringens* CNA has only been validated at the genetic level, but has not been tested or detected directly at the protein level in biological samples such as serum, feces, or jejunal digesta from NE-afflicted chickens, and chicken samples from farms with suspected NE outbreaks. In our previous studies, we developed a mouse monoclonal antibody (**mAb**)-based NetB sandwich enzyme-linked immunosorbent assay (**ELISA**) and validated its detectability of NetB in jejunal digesta, feces, and serum samples from NE-afflicted broiler chickens for the first time (Lee et al., 2020, 2021). Likewise, we used our newly developed CNA-specific sandwich ELISA to detect CNA protein in NE-afflicted chicken samples. In addition to testing CNA levels in NE-afflicted biological samples from the laboratory NE disease model, we also compared the CNA and NetB levels of jejunal digesta samples from commercial broilers in the United States. Our first hypothesis was that if the detection levels of CNA and NetB measured in NE-afflicted chicken samples from laboratory model were found to be significantly associated through correlation coefficient analysis, CNA and NetB were related under experimental NE conditions. The second hypothesis was that the detection levels of CNA and NetB were correlated through correlation coefficient analysis in unknown poultry field samples under the conditions where the first hypothesis was satisfied. If the levels of both CNA and NetB show a significant correlation in both conditions, CNA will be considered another important biomarker in the indirect diagnosis of NE outbreak as much as NetB, which is already widely accepted as a virulence factor of NE.

Therefore, the objective of this study was to first compare detectable CNA and NetB levels in biological samples from NE-afflicted chickens, and then evaluate jejunal digesta samples from various commercial farms in the United States using our newly developed mAb-based CNA- and NetB-specific sandwich ELISAs.

## MATERIALS AND METHODS

### Chickens, Experimental Design, and Biological Sample Collections

All experiments were conducted following a standard protocol approved by the Institutional Animal Care and Use Committee at Beltsville Agricultural Research Center (Protocol No. 17-027). One-day-old Ross 708 broilers were purchased (Longenecker's Hatchery, Elizabethtown, PA) and grown in the starter brooder units (Alternative Design Manufacturing and Supply, Inc., Siloam Springs, AR), and feed and water were provided ad libitum. All chickens were maintained in a room with temperature-controlled conditions. A total of 40 chickens were divided into 2 groups: uninfected negative control (**NC**) group and *Eimeria maxima* + *C. perfringens* dual-challenged (**CP**) group. Briefly, chickens in CP group were infected with *E. maxima* strain 41A (5,000 oocyst/chicken) by oral gavage on d 20 posthatch, followed by oral administration with *C. perfringens* strain LLY_Tpel17 (2 × 10^8^ colony forming unit; **cfu**/chicken) on d 24 (4-days post *E. maxima* infection, **dpi**). The chickens in the NC group received an equal volume of phosphate-buffered saline (**PBS**) and followed by administration of Brain Heart Infusion medium (**BHI**, BD Bacto, Sparks, MD) with oral gavage accordingly. For more effective NE development, all chickens were fed 18% low crude protein diet from d 0 to 24 and changed to 24% high crude protein diet on d 24 (Park et al., 2022, Table 1). Before sampling, NE lesion scores were primarily confirmed by 3 independent observers, and sampling was done on 15 out of 20 chickens in the CP group which showed clear NE lesion scores of at least 2 levels (Lee et al., 2011b). It was confirmed that the NC group chickens did not show any NE lesions. For sample collection, 15 chickens per treatment were euthanized by cervical dislocation on d 26 (6 dpi from the first *E. maxima* challenge), and serum, jejunal digesta, and feces were collected. For preparing sample analysis, sera were centrifuged at 1,500 × *g* for 10 min. Jejunal digesta and fecal sample were each mixed with the equal volume of PBS and then submitted to centrifugation at 2,500 × *g* for 15 min.

**Table 1:** Ingredient compositions of basal diets (as-fed basis, %).

Ingredients, %	Low protein diet, d 0–24	High protein diet, d 24–26
Corn	69.01	55.78
Soybean meal	23.99	37.03
Soybean oil	2.75	2.97
Dicalcium phosphate	2.00	1.80
Calcium carbonate	1.40	1.51
Common salt	0.35	0.38
Vitamin mixture^1^	0.20	0.22
Mineral mixture^2^	0.15	0.15
DL-Met	0.10	0.10
60% Choline chloride	0.05	0.06
Total	100.0	100.0
Calculated values, %		
Crude protein	18.00	24.00
Calcium	1.19	1.20
Available phosphorus	0.54	0.51
Lys	1.00	1.40
Met + Cys	0.65	0.80
ME, Mcal/kg	3.6	3.5

1 Vitamin mixture provided the following nutrients in kg of diet: vitamin A, 2,000 IU; vitamin D_3_, 22 IU; vitamin E, 16 mg; vitamin K, 100 µg; vitamin B_1_, 3.4 mg; vitamin B_2_, 1.8 mg; vitamin B_3_, 23.8 mg; vitamin B_5_, 8.7 mg; vitamin B_6_, 6.4 mg; vitamin B_7_, 170 µg; vitamin B_9_, 800 µg; vitamin B_12_, 13 µg.

2 Mineral mixture provided the following nutrients in kg of diet: Fe, 400 mg; Zn, 220 mg; Mn, 180 mg; Cu, 21 mg; Co, 1.3 mg; Se, 0.2 mg.

After centrifugation, supernatants of all biological samples were filtered through a 0.2 μm PTFE filter and stored at −70°C for further analysis.

### Field Sample Collections

**Figure 1:**
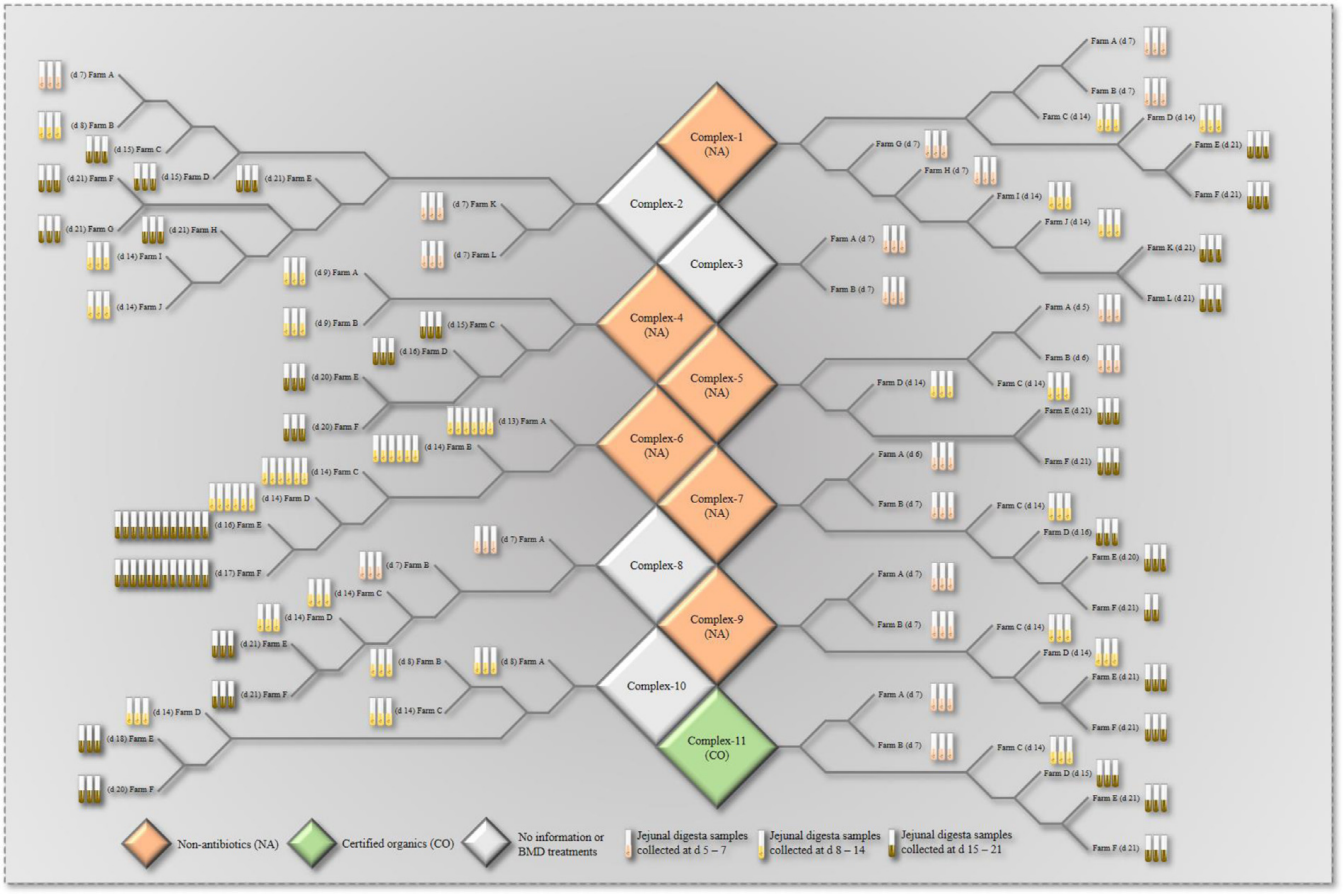
A map of jejunal digesta samples collected from commercial farms in the United States used in this experiment. A total of 251 jejunal digesta samples were randomly collected from 74 different commercial broiler farms in the United States. Seventy-four farms belong to 11 different poultry complexes from 3 poultry integrators. Of the 11 complexes, 6 are antibiotic-free (NA, “Complex-1, 4, 5, 6, 7, and 9”) farms, and 6 farms in Complex-11 used certified organics (Unknown information) (CO). Each farm provided 2 to 12 jejunal digesta samples. Each farm provided a sample on a day corresponding to one of the ages (d 5 to 21), and the age of the sample was indicated in 3 different colors in the figure.

All field jejunal digesta samples were collected from commercial broiler farms in the United States (Figure 1). All field samples were considered uninfected with NE. In brief, a total of 251 jejunal digesta samples were collected from 13 different time points during the early broiler production (d 5 to 21) from 74 different commercial farms of 11 different poultry complexes (Complex-1 to Complex-11) from 3 poultry integrators. A minimum of 2 to a maximum of 12 jejunal digesta samples were provided from each farm for quantitative analysis of CNA and NetB antigens. All samples were mixed with an equal volume of PBS and submitted to centrifugation at 2,500 × *g* for 15 min. After that, supernatants were collected and filtered through a 0.2 μm PTFE filter. The samples were stored at −70°C until analysis.

### Recombinant CNA Production

Recombinant CNA expressed in *Escherichia coli* was used for the current experiment. Briefly, the full-length coding sequence of the CNA (Appendix 1) gene was cloned into the pET-30a expression vector with an NH2-terminal polyhistidine epitope tag. Primers contained NdeI and HindIII restriction enzyme sites (645 amino acids with molecular weight = 72,480.5). Recombinant CNA/pET-30a was transformed into *E. coli* strain BL21 Star (DE3) cells (Life Technologies, Grand Island, NY). A single colony was inoculated with 1.0 mM isopropyl beta-D-thiogalactopyranoside (Amresco, Cleveland, OH). The recombinant CNA was purified using Ni-NTA His Bind Resin column (NOVAGEN, Darmstadt, Germany) and the concentration was determined by Bradford protein assay (Pierce Bradford Protein Assay Kit, Thermo Scientific, Rockford, IL). The molecular weight of CNA was confirmed using Coomassie bright blue stained SDS-acrylamide gels.

### Production of CNA mAbs

Mouse hybridomas secreting anti-CNA mAbs were developed using C57BL/6 mice by Genscript Biotech Inc. (Piscataway, NJ). Briefly, C57BL/6 mice (National Cancer Institute, Frederick, MD) were immunized biweekly by intraperitoneal and subcutaneous injections with 50 μg of purified *E. coli* CNA in Freund's adjuvant (Sigma-Aldrich, St. Louis, MO) (Lee et al., 2011a). Splenic lymphocytes were fused with mouse myeloma cell line SP2/0 (ATCC, Manassas, VA) and hybridomas selected in RPMI-1640 medium supplemented with hypoxanthine, aminopterin, and thymidine (all from Sigma-Aldrich, St. Louis, MO). Hybridomas secreting CNA mAbs were selected by an indirect ELISA based on high binding affinity. In brief, 96-well microtiter plates were coated with 1 µg/mL purified *E. coli* CNA in pH 7.4 PBS and overnight at 4°C. Plates were blocked with PBS containing 1% bovine serum albumin (**BSA**) and washed with PBS-Tween (**PBS-T**, pH 7.2 PBS + 0.05% Tween 20, Thermo Scientific, Rockford, IL). One hundred microliters of hybridoma culture supernatant was added into each well and the plate was incubated for 1 h at room temperature (**RT**) with agitation. After washing plates with PBS-T, bound mAbs were detected using horseradish peroxidase (**HRP**)-conjugated secondary antibody by 1: 10,000 dilutions (Peroxidase-AffiniPure Goat Anti-Mouse IgG, Jackson ImmunoResearch Inc., West Grove, PA). The plate was washed with PBS-T and 100 μL of 3,3’,5,5’-tetramethylbenzidine (**TMB**) substrate (Sigma-Aldrich, St. Louis, MO) was added, and TMB reaction was stopped with 2 M H_2_SO_4_. The 96-well plate was read by a microplate reader (Bio-Rad, Richmond, CA) with an optical density (**OD**) value at 450 nm (**OD_450_**). Ten hybridoma supernatants containing mAbs with antibody titers above 1:2,430 dilution factors were selected by limiting dilution and used for further characterization. The selected 10 mAbs (1D3, 2A5, 4B8, 6D4, 6D6, 6E4, 7H11, 9D5, 9G12, and 14A8) were purified from hybridoma supernatant by Protein G agarose chromatography, then the purified antibodies were biotinylated by biotinylation kit (EZ-Link Sulfo-NHS-Biotinylation Kit, Thermo Scientific, Rockford, IL) according to the manufacturer's instructions. Immunoglobulin isotypes were determined by mouse mAb isotyping kit (Mouse Monoclonal Antibody Isotyping Kit, Sigma-Aldrich, St. Louis, MO) according to the manufacturer's instructions.

### SDS Page and Western-Blot Analysis

CNA mAbs were mixed with an equal sample buffer (0.125 M Tris-HCl (pH 6.8), 4% SDS, 20% glycerol, 10% 2-mercaptoethanol, and 0.5% bromophenol blue) and heated for 5 min at 95°C. The mixture was resolved on a 15% SDS-polyacrylamide gel and electroblotted onto nitrocellulose (Immobilon-P, Millipore, Bedford, MA). The membrane was blocked with blocking buffer (Superblock Blocking Buffer, Thermo Scientific, Rockford, IL) and washed with PBS-T and incubated with CNA mAbs. The membrane was washed and bound mAbs were incubated with HRP-conjugated secondary antibody (1:10,000 dilution). Final product was visualized using an ECL substrate (Clarity Western, Bio-Rad, Hercules, CA) and detected using imaging tool (ChemiDoc Touch Imaging System, Bio-Rad, Hercules, CA).

### Detection of CNA and NetB by Antigen Capture Sandwich ELISAs

The mAbs obtained from 10 hybridomas were first screened with indirect ELISA for CNA, and the 8 mAbs with the highest avidity to CNA with no reactivity to the negative control antigen were finally selected. These 8 mAbs were used to select the best sandwich ELISA pairs for antigen capture and detection for CNA. Briefly, each CNA-binding mAb (10 μg/mL) in carbonate buffer (BupH Carbonate-Bicarbonate buffer packs, Thermo Scientific, Rockford, IL) 100 μL was coated in 96-well microplates for overnight at 4°C. The plate was washed with PBS-T for 2 times and blocked with 200 μL of blocking buffer followed by incubation at 37°C for 1 h. The blocking buffer was removed and 100 μL of the CNA antigen (1 μg/mL) with 0.1% BSA/PBS mixture was added into each well, and the plate was incubated for 2 h at RT. The plate was washed with PBS-T for 6 times and 100 μL of biotinylated detection CNA mAbs by 0.33 μg/mL were added into each well, and the plate was incubated for 1 h at RT. The plate was washed again with PBS-T 6 times and 100 μL of Avidin-HRP (A7419, Sigma-Aldrich, St. Louis, MO) (1:12,500) was added into each well, and the plate was incubated for 50 min at RT. After incubation, the plate was washed with PBS-T 6 times and developed with 100 μL of TMB substrate for 10 min, and the reaction of TMB was stopped by 100 μL of 2 M H_2_SO_4_ solution. The plate was read at OD_450_ by a microplate reader. Finally, only one set of optimal sandwich ELISA pair mAbs representing the highest OD value for CNA was selected. The selected optimal pairs for CNA were used to detect CNA in several chicken samples by the method of sandwich ELISA described above. For the detection of NetB antigen in chicken samples, we used NetB-specific sandwich ELISA that we developed with minor modifications (Lee et al., 2020). In brief, capture NetB mAbs (25C2-2) were mixed with carbonate buffer by 5 μg/mL and coated 100 μL in 96-well microplate for overnight at 4°C. The plate was washed with PBS-T 2 times, blocked with 200 μL blocking buffer, incubated at 37°C for 1 h. The solution was removed and 100 μL of NetB antigen with 0.1% BSA/PBS mixture by 0.5 μg/mL was added, and the plate was incubated for 2 h at RT. The plate was washed with PBS-T 6 times and 100 μL of HRP-conjugated detection NetB mAbs (4G11-2) mixture by 0.25 μg/mL was added and incubated for 1 h at RT. Finally, the plate was washed with PBS-T 6 times and developed with a TMB substrate of 50 μL for 5 min and the reaction was stopped by 100 μL of 2 M H_2_SO_4_ solution. The OD value was measured at OD_450_ by a microplate reader.

### Culture of *C. Perfringens* and DNA Genotyping

*C. perfringens* strains LLY_Tpel17 and Del-1 that were used in this experiment were taken from previous experiments (Li et al., 2017; Gu et al., 2019; Lee et al., 2020). *C. perfringens* strain LLY_Tpel17 (divided into 3 different batches, LLY_Tpel17a, b, and c) and Del-1 were initially grown anaerobically at 37°C in chopped meat glucose medium (Anaerobe Systems, Morgan Hill, CA) and then moved into BYC medium (3.7% BHI medium + 0.5% yeast extract, Fisher Scientific, Hampton, NH + 0.05% L-cysteine, Sigma-Aldrich, St. Louis, MO) with 0.1% sodium thioglycolate (Sigma-Aldrich, St. Louis, MO). Thereafter, single bacterial colonies were collected and separated and then cultured in BHI medium overnight at 41°C. The cultured BHI medium was then centrifuged at 12,000 × *g* for 10 min to obtain supernatants. The supernatants of both *C. perfringens* strains were tested for CNA and NetB levels with CNA- and NetB-specific sandwich ELISA, and pellets of each strain were collected for DNA extraction as described by Wilson (2001). PCR cycle for the CNA and NetB detection was as follows: 40 cycles for 1 min at 94°C and 2 min at 60°C, and 7 min at 72°C for the final extension. Products from PCR were analyzed with electrophoresis in 1.5% agarose gel containing 5 μg/mL of gel strainer (SYBR DNA Gel Strain, Invitrogen, Carlsbad, CA) and visualized by UV transillumination (ChemiDoc Touch Imaging System, Bio-Rad, Hercules, CA). The primer sequences and expected product sizes of CNA and NetB were described in Table 2.

**Table 2:** Primer sequences for PCR analysis.

Primer^1^	Primer sequence, 5′–3′	Product size, bp	References
CNA	F: GGTGGATGGGCAACATTTAC R: CCTTGCTTGGATTCACCAGT	183	Lepp et al., 2021
NetB	F: CGCTTCACATAAAGGTTGGAAGGC R: TCCAGCACCAGCAGTTTTTCCT	316	Park et al., 2015

1 CNA, collagen adhesion protein; NetB, necrotic enteritis B-like toxin.

### Statistical Analysis

All samples for the ELISA test were performed in triplicate, and statistical differences of paired observations were analyzed using Student *t* test. Unpaired *t* test with Welch's correlation was used for the field samples that do not assume equal variances. Pearson's correlation coefficient test was also used to interpret the relationship between CNA and NetB levels. All statistical analysis was conducted by RStudio software (R version 4. 2. 2, RStudio PBC, Boston, MA), and the significance between 3 or more treatments was determined using Tukey's honestly significant difference test. Statistical differences between sample means were considered significant if the *P* value <0.05.

## RESULTS

### Development of CNA-Specific Sandwich ELISA

**Figure 2:**
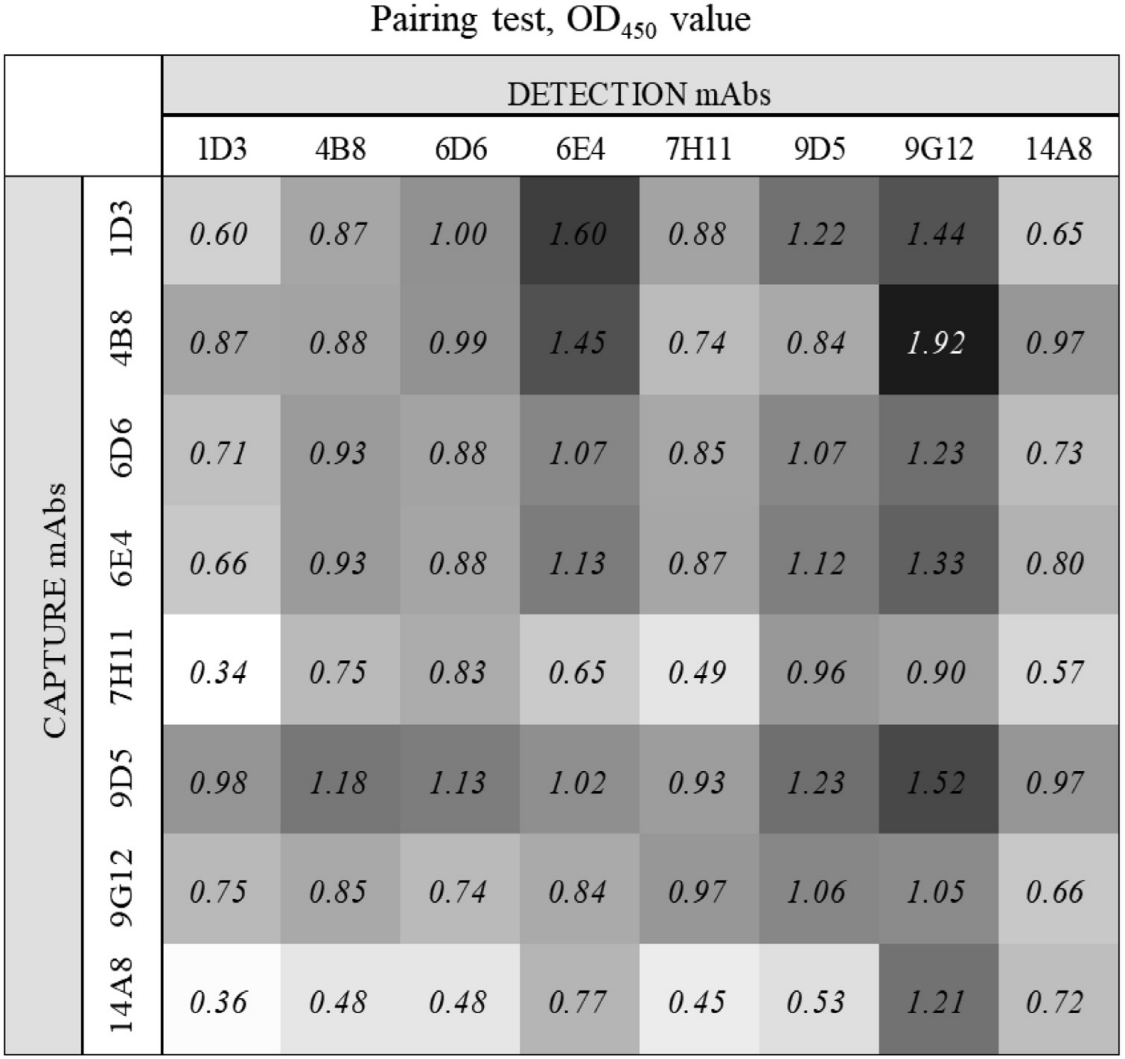
Pairing test of collagen adhesin protein (CNA) monoclonal antibodies (mAbs). Eight selected mAb clones were used to select the best pair of capture and detection mAbs for sandwich ELISA. All mAb previously tested their specificity to the CNA using indirect ELISA. All signals were detected in 450 nm optical density (OD_450_) by a microplate reader and darkest color in this figure considered as highest OD_450_ value (1.92; combination of 4B8 and 9G12).

**Figure 3:**
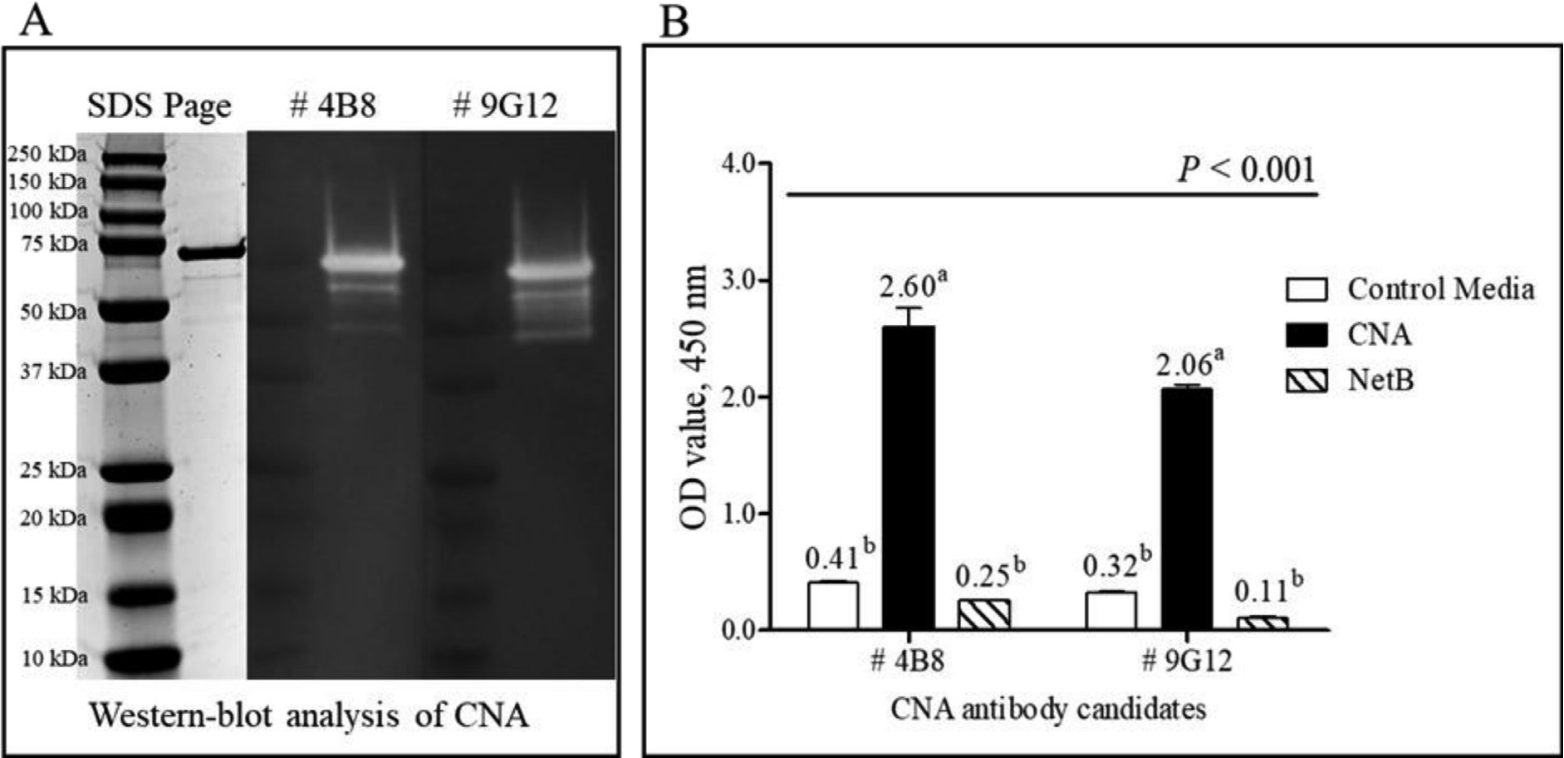
Western-blot analysis (A) and indirect ELISAs (B) to test antigen specificity of 2 collagen adhesin protein (CNA)-specific mAbs (4B8 and 9G12) from the paring test. For Western-blot analysis, the final product was visualized and detected using ChemiDoc Touch Imaging System. Both protein bands of 4B8 and 9G12 appeared at approximately 75 kDa. For indirect ELISAs, all signals were detected in 450 nm optical density (OD_450_) by a microplate reader (*n* = 3). ^a,b^Means in each bar with different superscripts are statistically different (*P* < 0.05).

Eight hybridomas secreting mouse mAbs with high avidity and specificity to CNA were selected to develop sandwich ELISA for CNA. The isotypes of most of CNA mAbs were IgG1, κ (1D3, 4B8, 6D6, 6E4, 7H11, 9D5, and 14A8) except 1 (9G12) which was IgG1, λ. In the paring assay, all 8 CNA mAbs were screened for capturing and detecting activities and 4B8 and 9G12 were selected as the best capture and detection mAb, respectively, based on their highest OD values (1.92) for binding to *E. coli*-expressed CNA (Figure 2). Western-blot analysis showed that the size of CNA which is recognized by both 4B8 and 9G12 mAbs was approximately 75 kDa protein (Figure 3A), and they did not react with NetB antigen. Indirect ELISA results showed that both CNA-specific mAbs (4B8 and 9G12) showed specific binding with CNA protein with high OD values (2.60 and 2.06) compared to control media (0.41 and 0.32, HBSS) and they did not detect NetB antigen (0.25 and 0.11) (*P* < 0.001, Figure 3B).

### Detection of CNA and NetB Antigens in Culture Supernatants of *C. Perfringens* Strains

**Figure 4:**
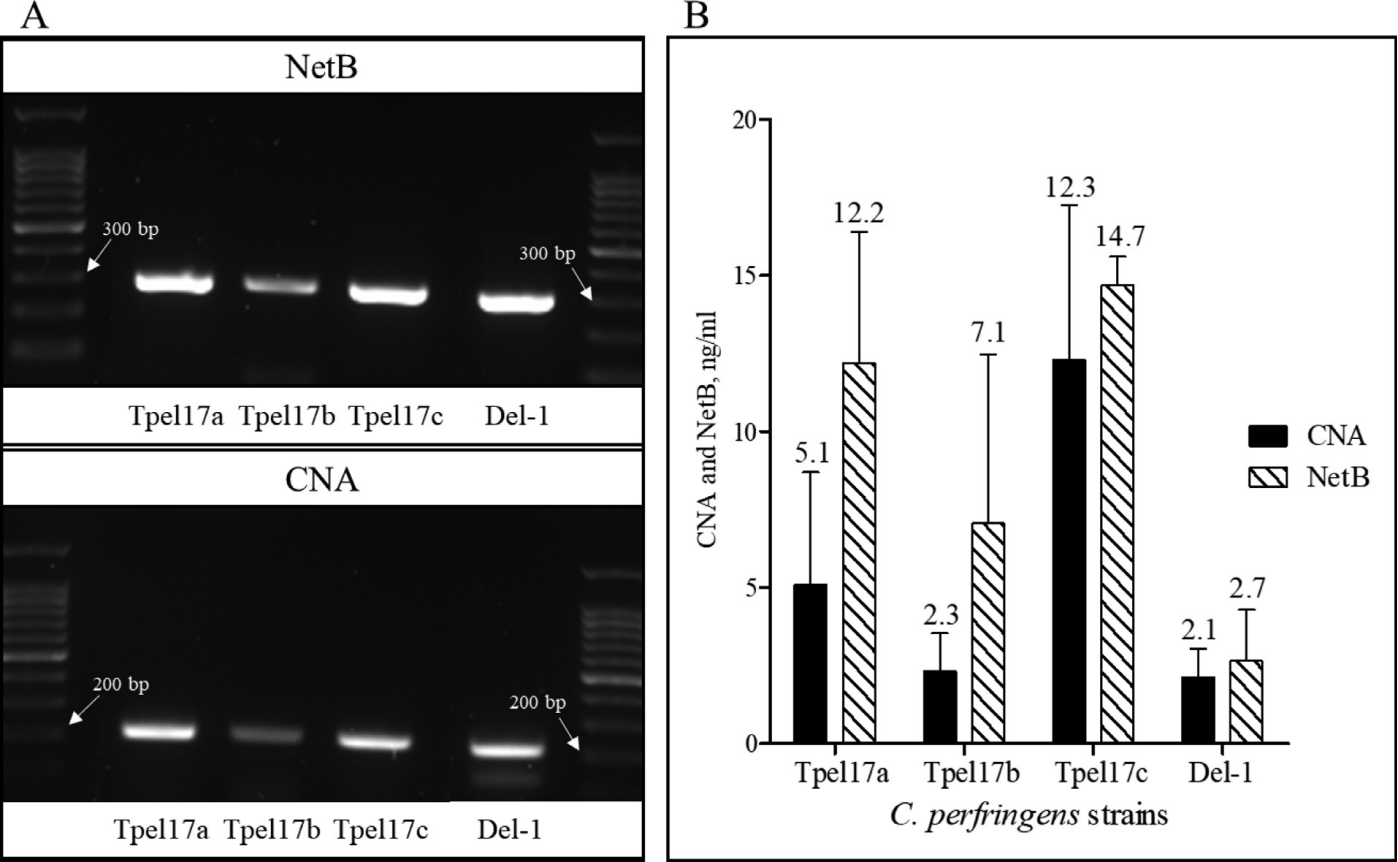
DNA genotyping result by PCR of collagen adhesin protein (CNA) and necrotic enteritis B-like toxin (NetB) genes in *C. perfringens* strains, LLY_Tpel17a-c and Del-1 (A). The bands of CNA and NetB appeared at approximately 200 and 300 bp, respectively. The product size of each CNA and NetB primer used in the current experiment were 183 and 316 bp, respectively (Table 2). The result of CNA and NetB-specific sandwich ELISAs of cultured *C. perfringens* supernatants showed that the LLY_Tpel17c strain had the highest levels of CNA and NetB titers (B). For sandwich ELISAs, all signals were detected in 450 nm optical density (OD_450_) by a microplate reader (*n* = 3).

To test cultural supernatants of randomly selected *C. perfringens* strains that we have in our collections, we carried out DNA genotyping. All 4 strains of *C. perfringens* that we tested showed positive bands of both CNA and NetB genes (Figure 4A). The product size of each CNA and NetB primer set used in the current experiment were 183 and 316 bp, respectively. After PCR test, bands of CNA and NetB were appeared at approximately 200 and 300 bp, respectively. Furthermore, both CNA and NetB proteins were detected in all the culture supernatants collected 24-h postincubation from the 4 strains of *C. perfringens*. Out of 4 strains of *C. perfringens* we tested, LLY_Tpel17c strain showed the highest levels of CNA and NetB (Figure 4B) proteins.

### CNA and NetB Detection in Biological Samples From NE-Afflicted Chickens

**Figure 5:**
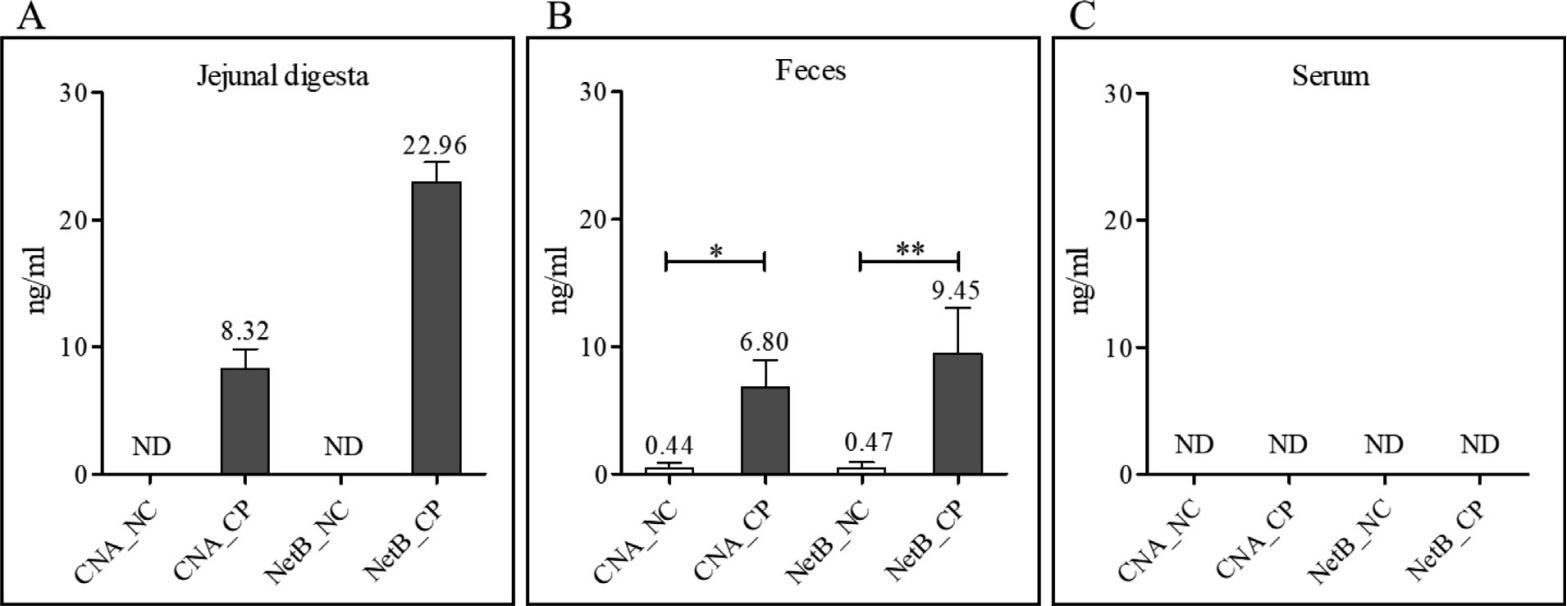
Detection of collagen adhesin protein (CNA) and necrotic enteritis B-like toxin (NetB) levels in jejunal digesta (*n* = 5; A), feces (*n* = 5; B), and serum (*n* = 10; C) in NE-afflicted broiler chickens using CNA- and NetB-specific antigen capture sandwich ELISAs. All biological samples were collected on 2 dpi (d 26) of *C. perfringens* infection in our laboratory NE disease model. The detection levels of CNA and NetB in fecal samples were significantly higher in CP groups compared to NC groups (CNA, *P* < 0.05; NetB, *P* < 0.01, B). The average detection levels of CNA and NetB in jejunal digesta were 8.32 and 22.96 ng/mL, respectively. The average detection levels of CNA and NetB in feces in CP group were 6.80 and 9.45 ng/mL, respectively, with minimum levels of detectable CNA (0.44 ng/mL) and NetB (0.47 ng/mL) in NC group. All signals were detected in 450 nm optical density (OD_450_) by a microplate reader. Abbreviations: CP, *E. maxima* and *C. perfringens* infection group; NC, noninfected control group; ND, not detected both CNA and NetB. *, *P* < 0.05; ^⁎⁎^, *P* < 0.01.

**Figure 6:**
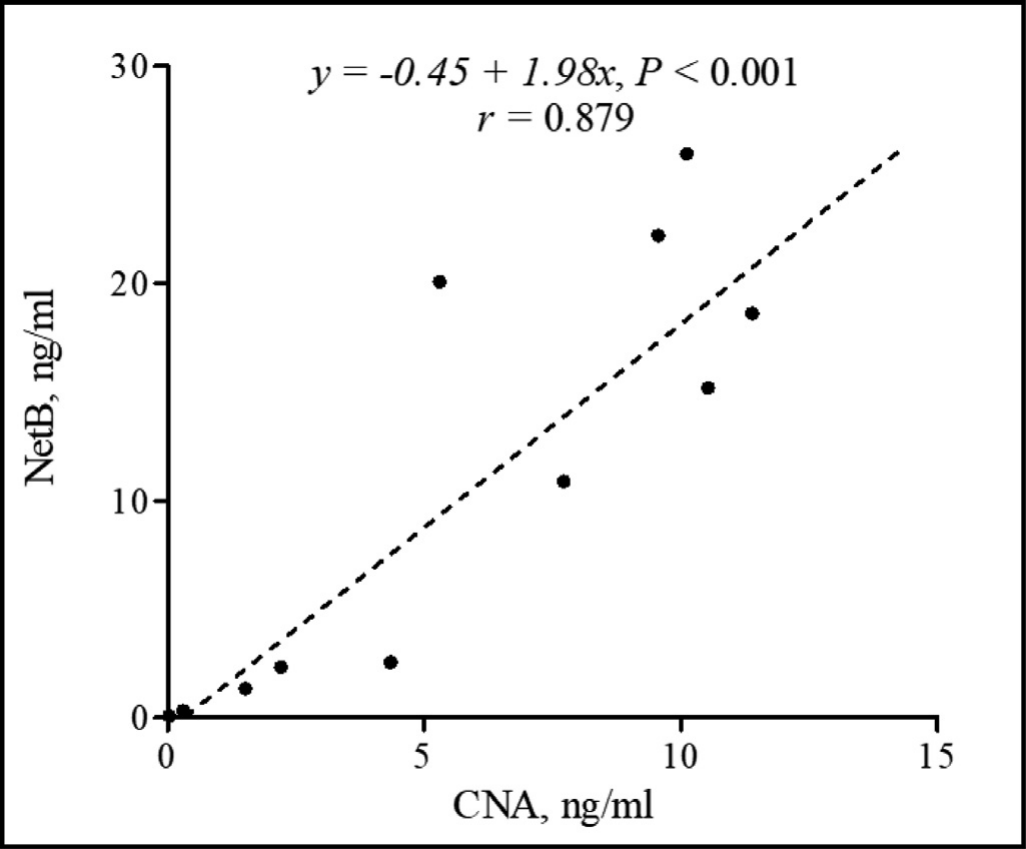
Pearson correlation coefficient between collagen adhesin protein (CNA) and necrotic enteritis B-like toxin (NetB) (*n* = 12) of all detected (>0 ng/mL of CNA and NetB) biological samples in broiler chickens which were collected in broiler chickens afflicted with our NE disease model. All values were measured by CNA- and NetB-specific sandwich ELISAs. The calculated correlation coefficient was expressed to be *r* value. The correlation between CNA and NetB in biological samples showed a strong positive correlation (*y* = −0.45 + 1.98*x, r* = 0.879, *P* < 0.001).

All of the NE-afflicted biological samples that we collected from chickens that were infected using a coinfection NE model (Lee and Lillehoj, 2021) were analyzed using new CNA- and NetB-specific antigen capture sandwich ELISAs. In the feces and jejunal digesta, CNA and NetB antigens were detected in the CP group with significantly higher levels of CNA and NetB titers than those detected in the NC group (Figure 5A and B). No CNA and NetB were detected in NC groups in jejunal digesta samples (Figure 5A). The average detection levels of CNA and NetB in NE-infected jejunal digesta were 8.32 and 22.96 ng/mL, respectively. The average detection levels of CNA and NetB in feces in CP group were 6.80 and 9.45 ng/mL, respectively (Figure 5B), with minimum levels of detectable CNA (0.44 ng/mL) and NetB (0.47 ng/mL) in NC group. The levels of CNA and NetB in fecal samples were significantly higher in CP groups compared to NC groups (CNA, *P* < 0.05; NetB, *P* < 0.01, Figure 5B). CNA and NetB were not detected in any groups of serum samples (Figure 5C). Comparing all biological samples containing CNA or NetB, there was a strong positive correlation between CNA and NetB by Pearson's correlation coefficient (*y* = −0.45 + 1.98*x, r* = 0.879, *P* < 0.001, Figure 6).

### CNA and NetB Detection in Jejunal Digesta Samples From Commercial Poultry Farms

**Figure 7:**
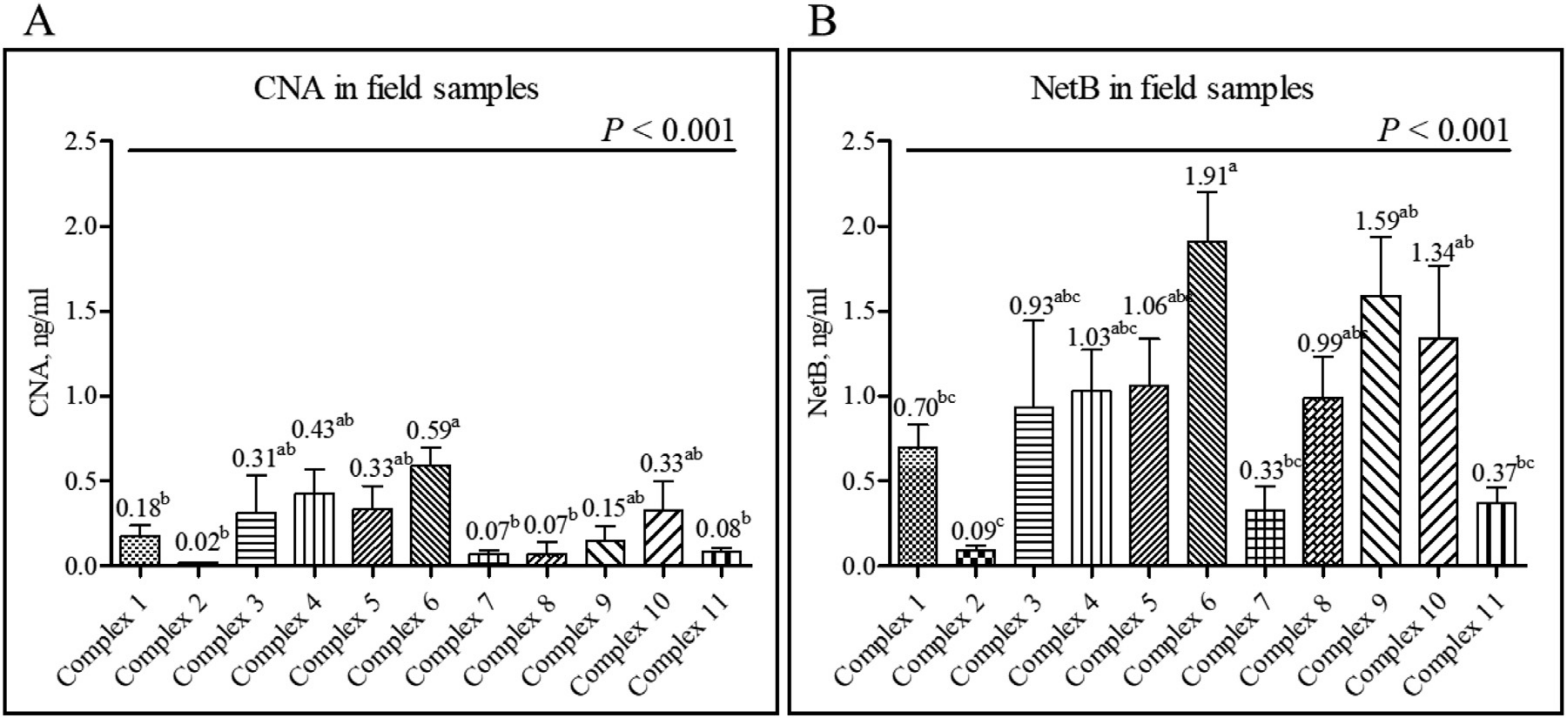
Detection of collagen adhesin protein (CNA) and necrotic enteritis B-like toxin (NetB) in the field jejunal digesta samples from 11 complexes in the United States using CNA- and NetB-specific sandwich ELISAs. The 11 complexes consist of 74 different farms, and each farm provided 2 to 12 jejunal digesta samples. “Complex-6” showed significantly increased CNA and NetB levels than “Complex-1, -2, -7, and -11” (*P* < 0.001; A and B). Detection ranges for CNA were from a minimum of 0.02 to a maximum of 0.59 ng/mL (A), whereas NetB were from a minimum of 0.09 to a maximum of 1.91 ng/mL (B). All signals were detected in 450 nm optical density (OD_450_) by a microplate reader (minimum *n* = 6, Complex 3; maximum *n* = 48, Complex 6). ^a–c^Means in each bar with different superscripts are statistically different (*P* < 0.05).

**Figure 8:**
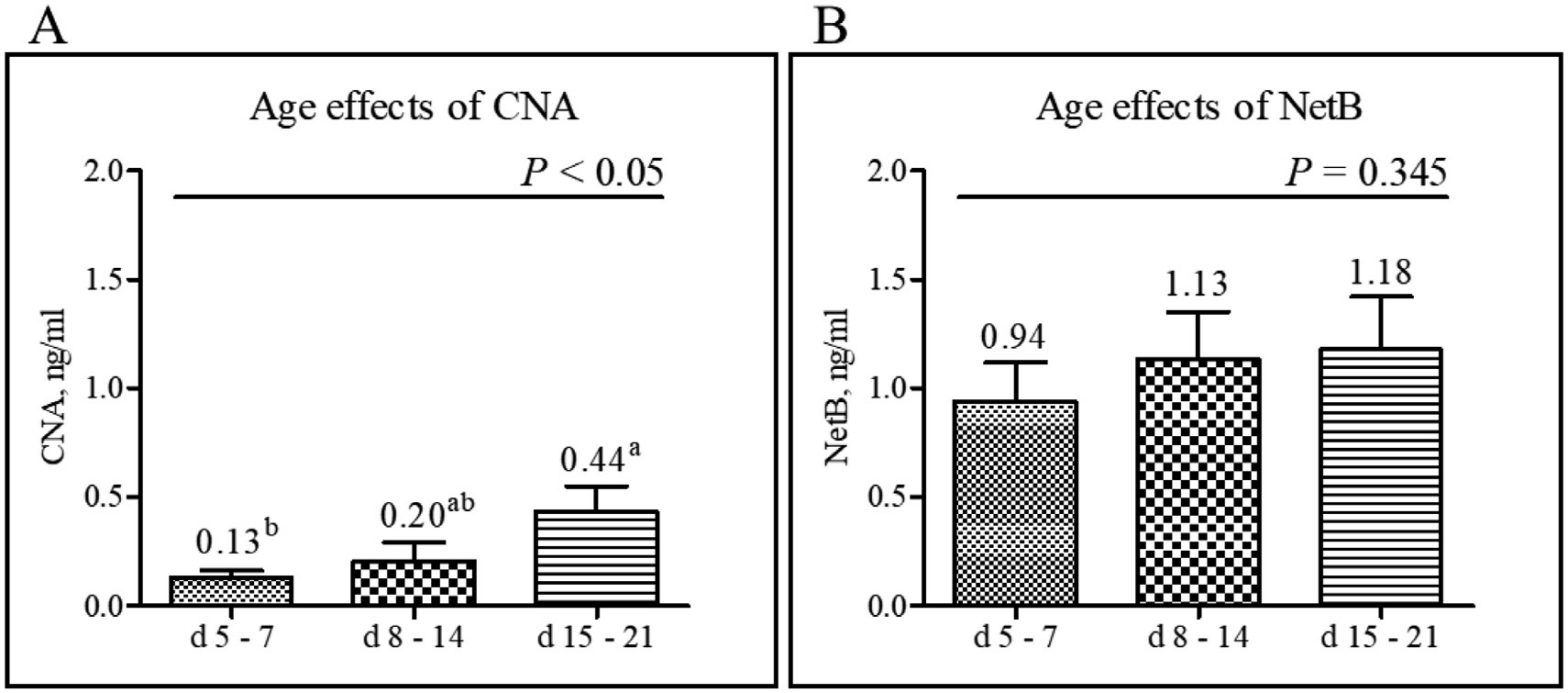
Detection of collagen adhesin protein (CNA) and necrotic enteritis B-like toxin (NetB) in the 108 randomly selected jejunal digesta (*n* = 36) of chickens of 3 different age groups (d 5–7, 8–14, and 15–21) using CNA- and NetB-specific sandwich ELISAs. The jejunal digesta samples collected from d 15 to 21 showed significantly increased CNA levels compared to those collected from d 5 to 7 (*P* < 0.05, A). When the d 5 to 7 and d 15 to 21 groups were compared, the average level of detected CNA increased by 238% from 0.13 to 0.44 ng/mL, while the level of NetB increased by 25% from 0.94 to 1.18 ng/mL. All signals were detected in 450 nm optical density (OD_450_) by a microplate reader. ^a,b^Means in each bar with different superscripts are statistically different (*P* < 0.05).

**Figure 9:**
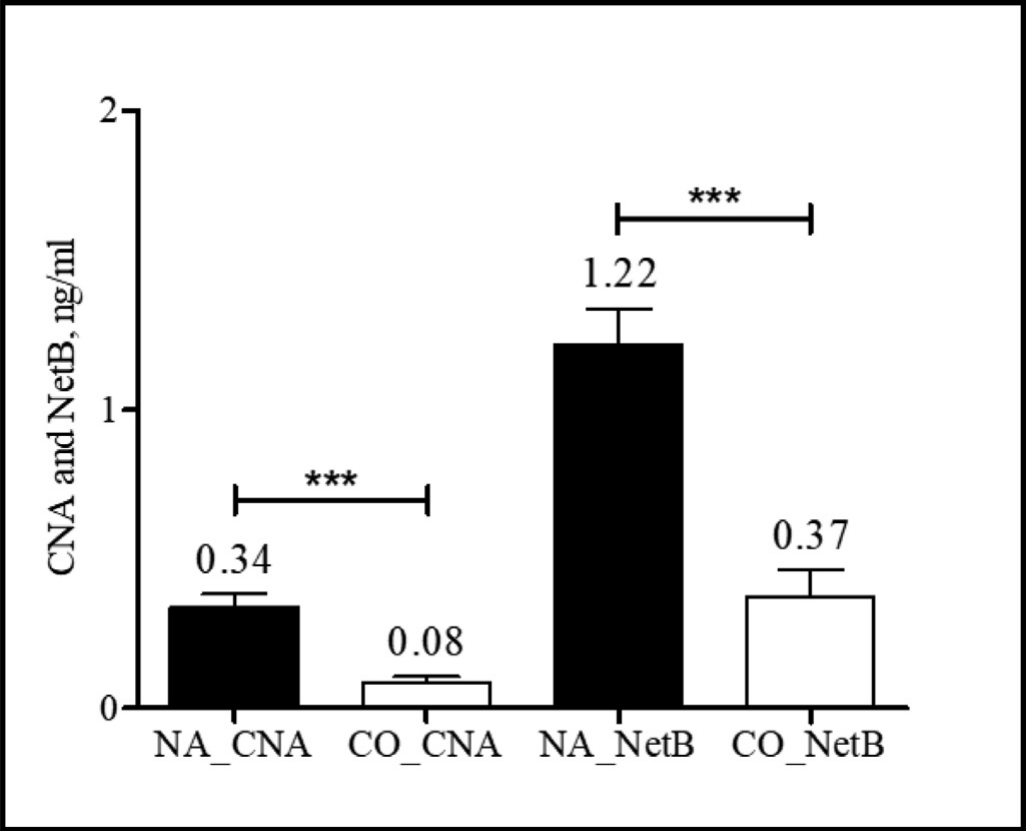
Detection of collagen adhesin protein (CNA) and necrotic enteritis B-like toxin (NetB) in jejunal digesta samples from antibiotic-free farms (NA, “Complex-1, 4, 5, 6, 7, and 9,” *n* = 155) and the farms used certified organics (CO, “Complex-11,” *n* = 18) using CNA- and NetB-specific antigen capture sandwich ELISAs. The levels of CNA and NetB were significantly decreased in the jejunal digesta taken from the CO farms compared to those from NA farms. All signals were detected in 450 nm optical density (OD_450_) by a microplate reader. ^⁎⁎⁎^, *P* < 0.001.

**Figure 10:**
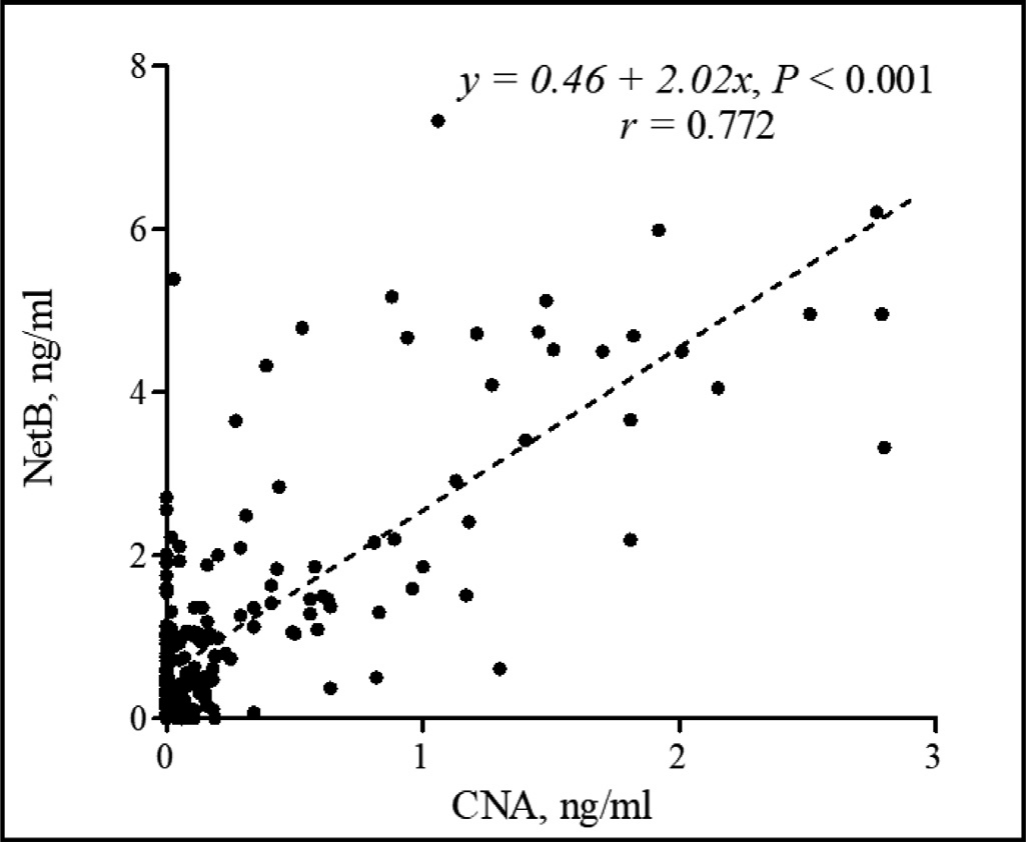
Pearson correlation coefficient between collagen adhesin protein (CNA) and necrotic enteritis B-like toxin (NetB) (*n* = 251) of jejunal digesta samples from commercial broiler farms in the United States. All values were measured by CNA- and NetB-specific sandwich ELISAs. The calculated correlation coefficient was expressed to be *r* value. The correlation between CNA and NetB in field samples showed a strong positive correlation (*y* = 0.46 + 2.02*x, r* = 0.772, *P* < 0.001).

A total of 251 field jejunal digesta samples from commercial farms in the United States were tested using CNA- and NetB-specific sandwich ELISAs. Detection levels for CNA ranged from a minimum of 0.02 to a maximum of 0.59 ng/mL (Figure 7A), whereas NetB ranges from a minimum of 0.09 to a maximum of 1.91 ng/mL (Figure 7B). Among the 11 poultry complex sources, “Complex-6” showed the highest CNA and NetB levels, and “Complex-2” showed the lowest level. For the CNA detection, “Complex-6” showed significantly increased level than “Complex-1, -2, -7, -8, and -11” (*P* < 0.001; Figure 7A). For the NetB detection, “Complex-6” also showed significantly increased NetB levels compared to “Complex -1, -2, -7, and -11” (*P* < 0.001, Figure 7B). Regardless of poultry complex, the jejunal digesta samples collected from d 15 to 21 showed significantly increased CNA levels compared to those collected from d 5 to 7 (*P* < 0.05, Figure 8A). In contrast, the level of NetB showed no significant differences among the 3 age groups even though detection level of NetB numerically increased with age (*P* = 0.345, Figure 8B). A comparison of CNA and NetB detection levels in jejunal digesta obtained from antibiotic-free (**NA**) farms (6 out of 11 complexes) and certified organics (**CO**) (Unknown information) used farms (1 out of 11 complexes), the average CNA and NetB levels in NA farms were 0.34 and 1.22 ng/mL, respectively (Figure 9). The average CNA and NetB detection levels in CO farms were 0.08 and 0.37 ng/mL, respectively. Significantly decreased levels of CNA and NetB levels were found in the jejunal digesta taken from the CO farms compared to the NA farms (*P* < 0.001, Figure 9). The Pearson's correlation coefficient between detection levels of CNA and NetB in 251 field jejunal digesta samples showed a strong positive correlation with each other (*y* = 0.46 + 2.02*x, r* = 0.772, *P* < 0.001, Figure 10).

## DISCUSSION

Although NE is a devastating enteric infection caused by *C. perfringens* that impacts the global poultry industry, there has not been a reliable early detection assay for NE disease outbreak in the commercial production system. The results of our study showed that anti-CNA antigen capture sandwich ELISA, together with anti-NetB sandwich ELISA that we previously developed (Lee et al., 2021), allows sensitive quantitative assessment of NE-associated virulence proteins in field samples from commercial poultry farms before 3 wk of age (5–21 d) when most field NE outbreaks occur. To demonstrate the efficacy of NE detection using these 2 sandwich ELISAs to detect NetB and CNA, we collected jejunal digesta samples from broiler chickens from several commercial poultry farms and conducted quantitative measurements for NetB and CNA antigens at different time points during the early broiler production period. The results clearly demonstrated that these new sandwich ELISAs can detect NE virulence factors starting from 5 d of the poultry production cycle with high sensitivity in jejunal digesta samples. This is the first description of mAb-based immunoassays that allow large field sample screening for NE-related biomarkers associated with NE outbreaks in commercial poultry farms posthatch.

NetB produced by *C. perfringens* type G has been identified as one of the main virulence factors in NE pathogenesis ( Keyburn et al., 2010; Rood et al., 2018). There has not been a sensitive immunoassay to make a quantitative assessment of NetB in NE-afflicted poultry. In this study, we used the NetB-specific antigen capture sandwich ELISA (Lee et al., 2020, 2021), and a newly developed CNA-specific antigen capture sandwich ELISA to identify potential NE susceptible flocks in commercial broiler farms during early broiler production. These 2 antigens of *C. perfringens* are important biomarkers of NE infection since NetB is a primary toxin associated with NE, and CNA is involved in the host cell invasion of *C. perfringens* to attach to ECM to maximize the effectiveness of toxins (Wade et al., 2016). CNA protein is present in some pathogenic strains of *C. perfringens* and is known to play an important role in NE pathogenesis by providing increased adhesion to collagen especially in the damaged intestine (Wade et al., 2015). In spite of the important role of CNA in NE infection and in NE pathogenesis, there has not been any quantitative assay to detect them in NE-infected samples. As a result of quantitative analysis, the detection levels of CNA and NetB in various samples from NE-afflicted chickens and samples from commercial farms using CNA- and NetB-specific sandwich ELISAs, there were high levels of the correlation between the CNA and NetB.

Antigen capture sandwich ELISA for detecting CNA of *C. perfringens* was developed after the extensive screening of highly specific murine mAbs that we developed using *C. perfringens* CNA antigen. By selecting 4B8 and 9G12 mAbs as capture and detection antibodies, respectively, this new CNA-specific antigen capture sandwich ELISA showed highest specificity and avidity to use in detecting NE-associated biomarkers in field samples. To verify the specificity and usefulness of this ELISA for detecting *C. perfringens* CNA, target antigen specificity was confirmed by Western-blot analysis using the 2 mAbs which detected around 75 kDa *C. perfringens* antigen which is similar in size to that of CNA by SDS-PAGE. Indirect ELISA also confirmed that 2 CNA mAb candidates, 4B8 and 9G12, specifically bound to CNA from CNA^+^ *C. perfringens* strains, LLY_Tpel17 and Del-1 (Lee and Lillehoj, 2021; Lee et al., 2021). The cultural supernatant from the *C. perfringens* strain that we used in our NE model also showed CNA^+^ and NetB^+^ (Lee et al., 2021). Furthermore, we verified that the *C. perfringens* strains LLY_Tpel17 and strain Del-1 which induce NE in our laboratory NE model, both contained bands corresponding to these 2 antigens of *C. perfringens*. In addition to DNA genotyping analysis, the bacterial culture supernatants of *C. perfringens* strain LLY_Tpel17 and Del-1 showed reactivity with CNA- and NetB-specific mAbs in 2 sandwich ELISAs confirming CNA and NetB secretion by these 2 *C. perfringens* strains.

To validate the detection sensitivity of CNA-specific sandwich ELISA, biological samples including serum, jejunal digesta, and feces were collected from broiler chickens which were infected with *E. maxima* and *C. perfringens*, with 4 d apart, which was outlined in our NE disease model (Lee et al., 2020, 2021) using *E. maxima* strain 41A and *C. perfringens* strain LLY_Tpel17. After that, it was confirmed that there was a clear difference in NE lesion scores between the CP group and the NC group (data not shown). The detected CNA in jejunal digesta and feces were numerically lower than the detected levels of NetB. However, both CNA and NetB showed very high levels of detection in the CP group compared to the NC group. This clearly showed that our CNA-specific sandwich ELISA is sensitive enough for CNA detection in biological samples from NE-afflicted broiler chickens. In the current experiment, the source, time (age of chickens and dpi), and strain of *C. perfringens* for sampling jejunal digesta and feces were all different from our previous NetB-related experiment (Lee et al., 2020, 2021), but there was no problem with their detection ability. However, both CNA- and NetB-specific sandwich ELISA did not detect any CNA and NetB in serum samples which were collected at 2-days post-NE. This result is the same as in our earlier report by Lee et al. (2021) and showed the absence of NetB antigen from all serum samples collected under various challenge conditions (Li et al., 2017; Hofacre et al., 2019). The correlation coefficient analysis of all biological samples from NE-afflicted chickens with detectable (both CNA and NetB levels are higher than 0) CNA and NetB levels showed a strong positive correlation between CNA and NetB levels. This high correlation between CNA and NetB suggests that both antigens have a positive relationship under NE conditions and could be used as reliable biomarkers of NE disease.

To validate the usefulness of the anti-CNA sandwich ELISA against NE antigens, jejunal digesta samples were obtained from randomly selected broiler chickens from various commercial poultry farms located in the United States during early poultry production cycle. Analysis of a total of 251 jejunal digesta samples revealed that CNA was detected in the range of 0.02 to 0.59 ng/mL and NetB in the range of 0.09 to 1.91 ng/mL with average detectable levels of CNA and NetB being 0.23 and 0.94 ng/mL, respectively. The detection levels of CNA and NetB in biological samples from chickens infected using our laboratory NE model (Lee et al., 2020) were clearly higher (the average CNA and NetB levels in jejunal digesta, 8.32 and 22.96 ng/mL, respectively) than those observed in the field samples from commercial broiler farms. The jejunal digesta samples collected from the experimental NE were taken from chickens previously showing clear NE lesions (Lee et al., 2011b), and the lowest detection levels of CNA and NetB in these samples were 5.30 and 20.70 ng/mL, respectively. However, higher levels are usually obtained from chickens that show clear NE lesions. But in most commercial farms, chickens with subclinical NE may show lower levels of CNA and NetB antigens. The highest CNA and NetB levels that were detected in the samples from commercial farms in this study were 2.8 and 7.36 ng/mL, respectively. Although we could not do any correlation studies between the levels of CNA and NetB antigens and the severity of NE in field samples since there was no information concerning the NE status of commercial chickens from which these samples were obtained, the results of this study may indicate that our CNA- and NetB-specific sandwich ELISAs offer sensitive detection of NE virulence factors in various samples from commercial poultry farms earlier than 21-days posthatch before major NE outbreak occurs.

Even when the jejunal digesta samples from each farm were combined and averaged to compare with data from other complexes, the detection levels of CNA and NetB were very similar. For example, the farms in “Complex-2” showed the lowest average detection levels of both CNA and NetB (0.02 and 0.09 ng/mL) whereas the farms in “Complex-6” showed the highest average detection levels (0.59 and 1.91 ng/mL). In addition, similar detection patterns of CNA and NetB levels among different farm complexes can be explained as a result of a significant increase in the average detection levels of CNA and NetB in “Complex-6” than “Complex-1, -2, -7, and -11.” However, unlike most complexes showing similar patterns for CNA and NetB detection, the samples in “Complex-9” showed the detected average NetB level being the second highest (1.59 ng/mL) among all complexes, but the average CNA detection level was the seventh highest (0.15 ng/mL). This could be due to the differences among the *C. perfringens* strains that may exist on the farms at different locations. Unlike the CNA^+^ and NetB^+^ *C. perfringens* LLY_Tpel17 laboratory strain that we used in our study to produce NE-afflicted biological samples, *C. perfringens* strains which are naturally present in different poultry farms are likely to vary in their pathogenicity and virulence depending on the history of NE outbreak, the feeding practice, and the use of antibiotics. Moreover, it is possible to speculate that the pathogenicity of *C. perfringens* strains which are associated with each farm may be associated with the presence of CNA, NetB, or other toxins as previously described (Wade et al., 2016; Lee and Lillehoj, 2021).

A total of 108 jejunal digesta samples were randomly collected and classified into 3 different age groups: d 5 to 7, d 8 to 14, and d 15 to 21. The average detected levels of CNA and NetB were lowest in the d 5 to 7 (CNA, 0.13 ng/mL; NetB, 0.94 ng/mL) group with the highest level of detection in the d 15 to 21 (CNA, 0.44 ng/mL; NetB, 1.18 ng/mL) group. As with our previous results, CNA and NetB levels were higher in the d 15 to 21 group, possibly due to higher *C. perfringens* levels in the jejunal digesta in older broiler chickens. This result is similar to a previous study that showed that *C. perfringens* levels were 10 times higher in the ileum of chickens raised without antibiotics at d 14 or d 21 than d 7 (Knarreborg et al., 2002). When the d 5 to 7 and d 15 to 21 groups were compared, the average level of detected CNA increased by 238% from 0.13 to 0.44 ng/mL, whereas the level of NetB increased by 25% from 0.94 to 1.18 ng/mL. The detected CNA level of jejunal digesta samples in the d 15 to 21 group was significantly higher than those of d 5 to 7 group. These results indicate that CNA-specific sandwich ELISA has higher sensitivity to detect CNA proteins in the jejunal digesta samples from commercial farms compared to the NetB-specific sandwich ELISA. Although the detection ability of NetB did not significantly differ by the age groups, NetB-specific sandwich ELISA demonstrated higher overall detection sensitivity than CNA-specific sandwich ELISA regardless of age. Currently, higher disease outbreak has been associated with antibiotic-free production (Hofacre et al., 2018). Thus, it will be advantageous to detect both CNA and NetB either in jejunal digesta or feces in the early stages of the chicken production cycle before it is too late for treatment to reduce NE-associated economic losses (Lee et al., 2020; Lee and Lillehoj, 2021). Although early detection of NetB and CNA in newly hatched chickens may indicate local contamination of eggs or in-embryo contamination, careful survey of commercial farms for CNA and NetB will enable the timely application of appropriate prevention/therapeutic strategies to reduce losses due to NE.

Antibiotic-free production of commercial poultry has been associated with increased incidence of NE (Fancher et al., 2020), with many current commercial poultry productions becoming more dependent on using antibiotic-alternative feed additives. To evaluate the efficacy of CNA- and NetB-specific sandwich ELISAs for NE detection in antibiotic-free farms, jejunal digesta samples from NA farms and CO farms were evaluated. The detection levels of CNA and NetB in jejunal digesta from NA farms were significantly increased than those from CO farms. Decreased detection levels of CNA and NetB are likely caused by mitigated NE response associated with using organic materials as antibiotic alternatives (Yitbarek et al., 2012; Pham et al., 2020; Sun et al., 2020). However, in this experiment, limited information on CO and limited number of samples that we obtained makes it difficult to compare, and further studies are needed to determine the effects of antibiotic-free poultry production on NE disease outbreak that may correlate with the levels of CNA and NetB. The correlation coefficient between CNA and NetB detection in farm samples showed a strong positive correlation between CNA and NetB. Interestingly, unlike the correlation coefficient in CNA and NetB detection in biological samples from the experimental NE, randomly collected jejunal digesta samples from naïve chickens of commercial farms, also showed very similar strong positive correlations. Further in-depth analysis using a larger number of field samples with detailed management information could enhance our understanding of the interaction between CNA and NetB in *C. perfringens-*induced NE.

In conclusion, we successfully demonstrated that the newly developed mAb-based antigen capture sandwich ELISA for NE-associated antigens is specific and allows quantifiable antigen detection in the field samples, and when used with the NetB-specific sandwich ELISA, enhances early identification of NE-susceptible broiler chickens in commercial farms. The detection of CNA was successful in biological samples from the experimental NE infection and also in unknown samples from commercial poultry farms in the United States. Using CNA- and NetB-specific sandwich ELISA, preferentially high positive correlation was successfully verified in chicken samples from experimental NE. Furthermore, CNA and NetB levels still showed a significant positive correlation in commercial farm samples.

The use of these antigen-capture sandwich ELISAs will facilitate early detection of NE-associated biomarkers in disease susceptible poultry populations in commercial farms enhancing effective management of NE in commercial poultry production. The current study is the first scientific report showing the utilization of the CNA-specific sandwich ELISA on NE biomarker detection in commercial broiler chickens.

## Appendix Supplementary materials

Image, application 1
