# Supplementary figures and images for: Collagen adhesin protein and necrotic enteritis B-like toxin as biomarkers for early diagnosis of necrotic enteritis in commercial broiler chickens
Source: Poult Sci. 2023 Mar 14;102(6):102647. doi: 10.1016/j.psj.2023.102647 (PMC10139936; doi:10.1016/j.psj.2023.102647)

**APPENDIX**

Appendix 1: full-length protein sequence of CNA


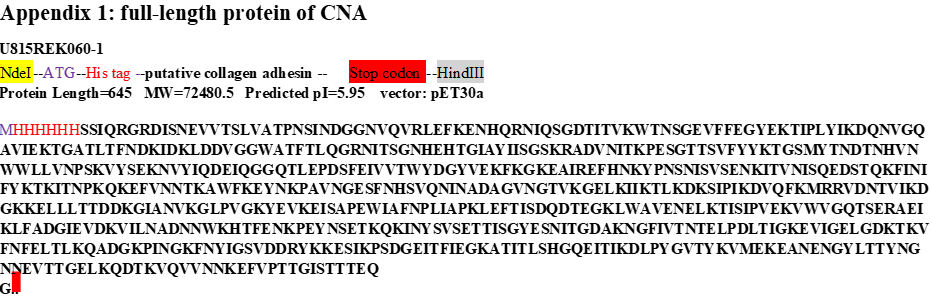

Supplement: Supplementary file 1 [file mmc1.docx]
